# Supplementary material for: Targeted removal of epigenetic barriers during transcriptional reprogramming
Source: Nat Commun. 2019 May 9;10:2119. doi: 10.1038/s41467-019-10146-8 (PMC6509258; doi:10.1038/s41467-019-10146-8)
Supplement: Supplementary file 1 — Supplementary Information [file 41467_2019_10146_MOESM1_ESM.pdf]

Targeted removal of epigenetic barriers during transcriptional reprogramming

Baumann et al.

Supplementary Information

Supplementary Figure 1

Baumann et al.

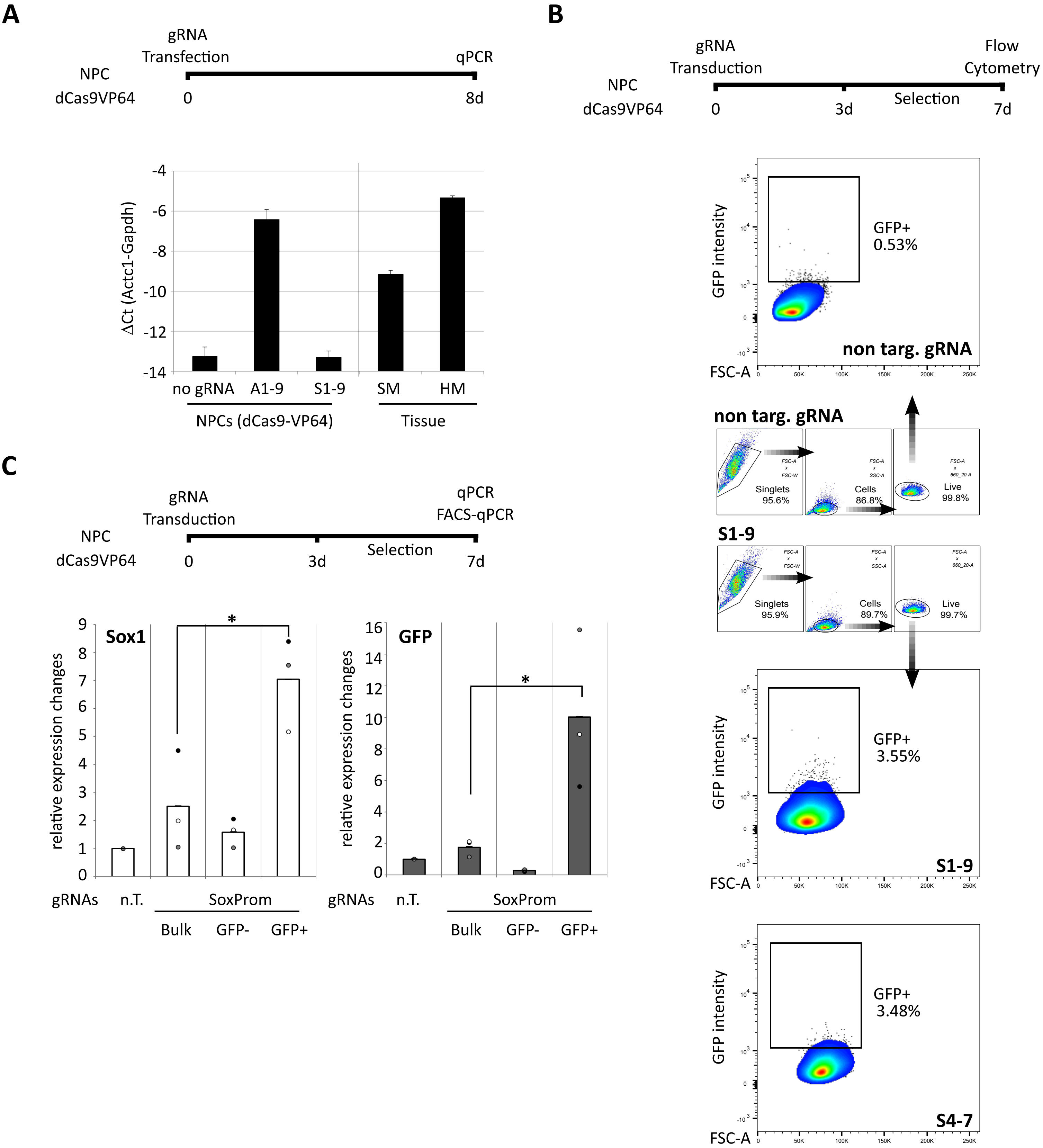

Supplementary Figure 1:

(A) Significant induction of *Actc1* in NPCs using transcriptome engineering. ΔCt values (Ct:*Actc1* – Ct:*Gapdh*) indicative of mRNA levels are shown for NPCs with and without *Actc1* induction using dCas9-VP64 and compared to ΔCt derived from heart (HM) and skeletal muscle tissue (SM) of 12-month-old mice, in which *Actc1* is naturally expressed. Data shown as the mean and standard error of the mean of n = 3 biological replicates.

(B) Flow cytometry reveals a small population of responsive cells. Non-targeting gRNAs were transduced into $Sox1^{GFP}$ NPCs, and cells were selected for gRNA expression. Flow analysis of cells reveals that some but not all cells respond to transcriptional activation with higher GFP fluorescence. Different gRNA pairs (S1-9, S4-7) activate comparable fractions of cells (see also Figure 1D), while cells transduced with non-targeting gRNAs resemble non-transduced cells. gRNA lentiviruses were transduced into $Sox1^{GFP}$ NPCs (MOI = 4), and cells were selected for gRNA expression.

(C) GFP and *Sox1* mRNA are elevated in cells sorted from the GFP-positive gate. $Sox1^{GFP}$-positive and -negative cells were sorted from the dCas9-VP64 activated population; mRNA levels of GFP and Sox1 were quantified in unsorted (n.T., non-targeting; Bulk, unsorted cells transduced with SoxProm gRNAs) and sorted populations. The significantly higher mRNA levels in the GFP-positive population compared to the Bulk population indicate an enrichment of responsive cells. Data shown as independent biological replicates, as well as the mean (bar graphs); * $p < 0.05$ in Student's t-test; n = 3 biological replicates, performed on different days in different clonal lines.

Supplementary Figure 2 **Baumann et al.**

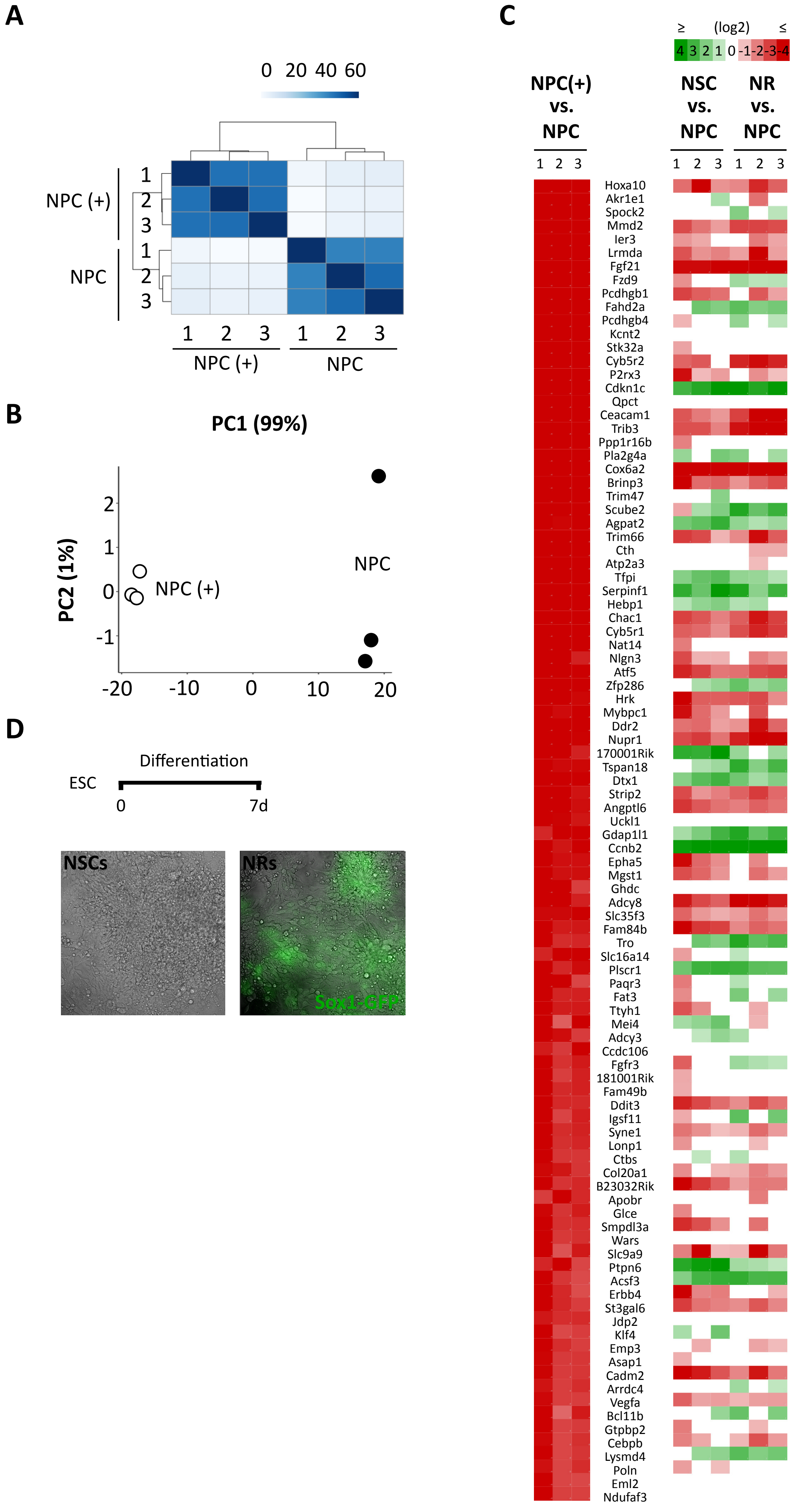

Supplementary Figure 2:

(A+B) RNAseq reveals significant changes in gene expression patterns after Sox1 upregulation. Heatmap and PC analysis of the transcriptomes of *Sox1*^{GFP}-positive NPCs (+) and controls (NPCs); two clusters arise, separating the two populations.

(C) Heatmap comparing gene expression changes of SoxI-positive (+) and -negative NPCs, NSCs and NRs. Log2 fold changes of the 100 most significantly downregulated genes in Sox1-positive cells are shown for all replicates. Data were collected from three biological replicates each.

(D) Exemplary pictures of NSCs and NRs after 7 days of neural differentiation from ESCs.

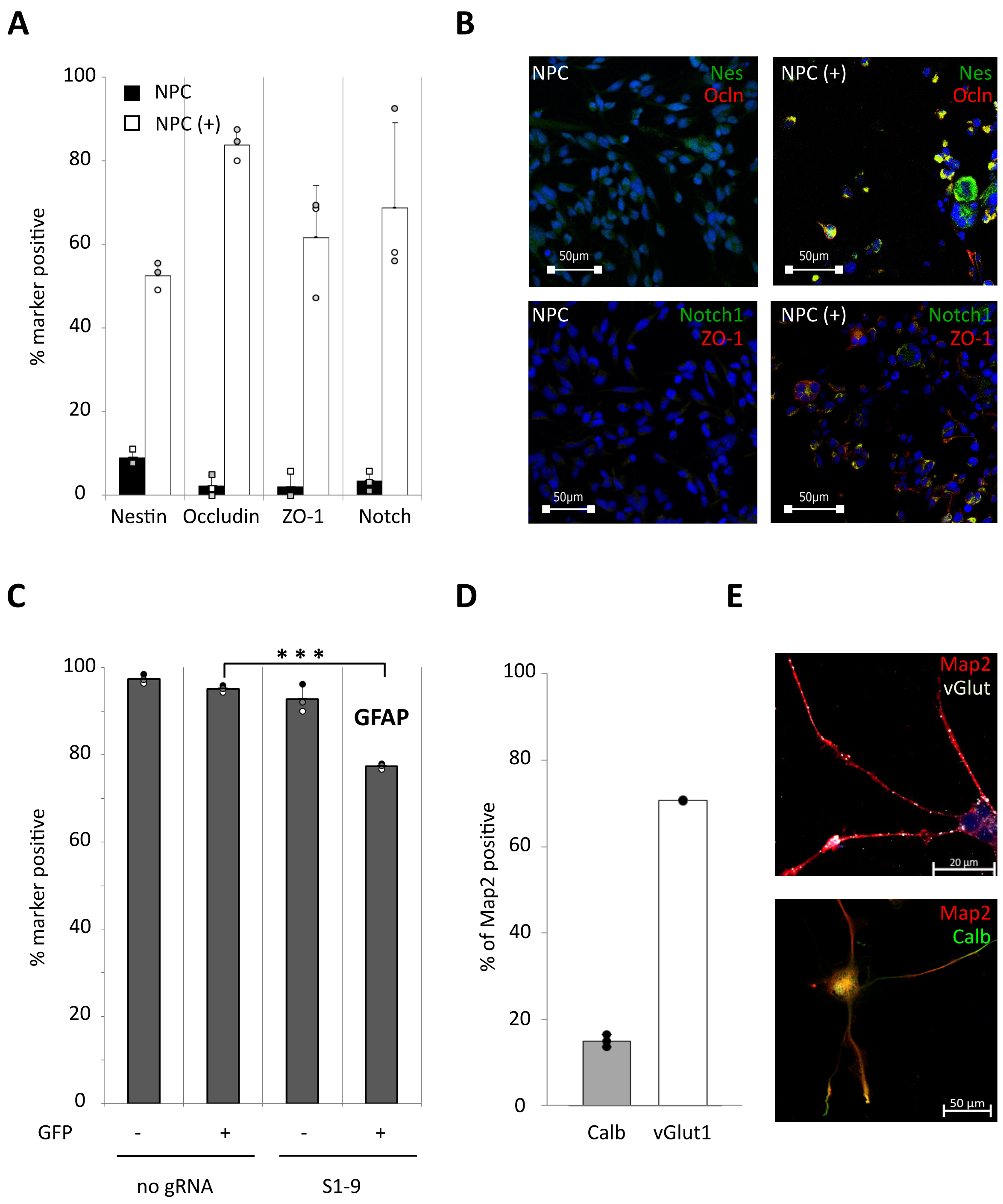
A
100
80
60
40
20
0
% marker positive
NPC
NPC (+)
Nestin
Occludin
ZO-1
Notch
B
NPC
Nes
Ocln
NPC (+)
Nes
Ocln
50µm
NPC
Notch1
ZO-1
NPC (+)
Notch1
ZO-1
C
* * *
GFAP
% marker positive
GFP
-
+
-
+
no gRNA
S1-9
D
% of Map2 positive
Calb
vGlut1
E
Map2
vGlut
20 µm
Map2
Calb
50 µm

Supplementary Figure 3:

(A+B) Sox1-positive NPCs express neuroepithelial markers. NPCs transduced with S1-9 gRNAs and sorted for *Sox1*^{GFP} expression were immunostained for the neuroepithelial markers Ocln and Zo-1, as well as for the neural stem cell markers Nestin and Notch1. *Sox1*^{GFP}-positive cells express these markers more frequently. Data shown as the mean + SEM. n = 3 biological replicates, performed on different days in different clonal lines.

(C) Differentiation reveals changes in the potency of Sox1-positive NPCs. After gRNA transduction, GFP-positive and -negative cells were sorted from both control (no gRNA) and activated (S1-9) populations and differentiated for 7 days; the number of cells positive for the glial marker Gfap decreased significantly in differentiating *Sox1*^{GFP}-positive cells; *** $p < 0.001$ in Student's t-test; n = 3 biological replicates, performed on different days in different clonal lines.

(D) The majority of neurons differentiated from Sox1-positive NPCs have glutamatergic features. *Sox1*-targeting gRNAs (S1-9) were transduced into *Sox1*^{GFP} NPCs. *Sox1*^{GFP}-positive cells were sorted for GFP expression 7 days later and differentiated for 21 more days to allow neurons to mature. Immunocytochemistry for neuronal subtype specific markers reveals that approximately 70% of Map2-positive neurons were also positive for vGlut1, while approximately 15% showed Calbindin expression. Data shown as the mean and standard error of the mean of n = 3 biologic replicates, performed on different days in different clonal lines.

(E) Mature neurons differentiated form *Sox1*^{GFP} positive NPCs. Images show examples of three vGlut1/Map2 double-positive neurons (top) and a Calbindin/Map2 double-positive neuron (bottom).

Supplementary Figure 4 Baumann et al.

A

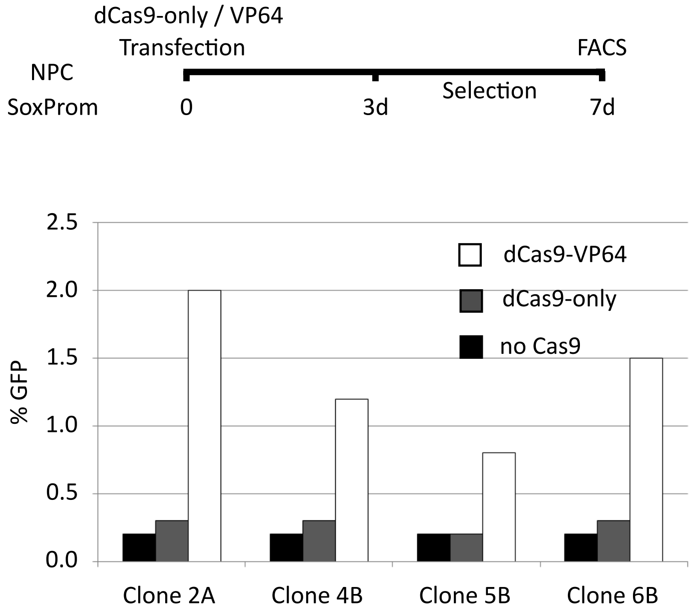

B

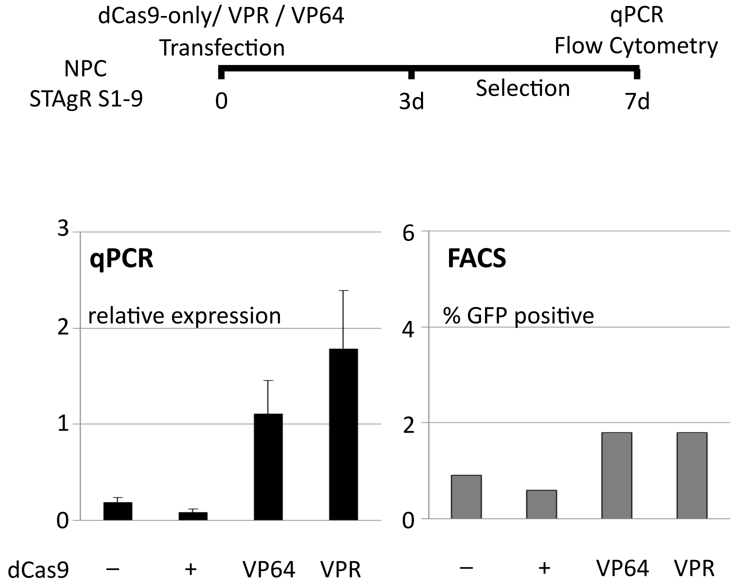

Supplementary Figure 4

(A) Clonal gRNA expressing NPC lines indicate no drastic impact of gRNA transduction on the responsiveness to *Sox1* trans-activation. dCas9-VP64 or only dCas9 were transfected into different clonal lines, stably expressing *Sox1* targeting gRNAs (SoxProm). Flow cytometry shows that no clonal line failed to respond entirely, while neither did one responded in a high proportion, indicating that gRNA expression and delivery might be negligible in this context.

(B) Biological replicate for data shown in Figure 3 D.

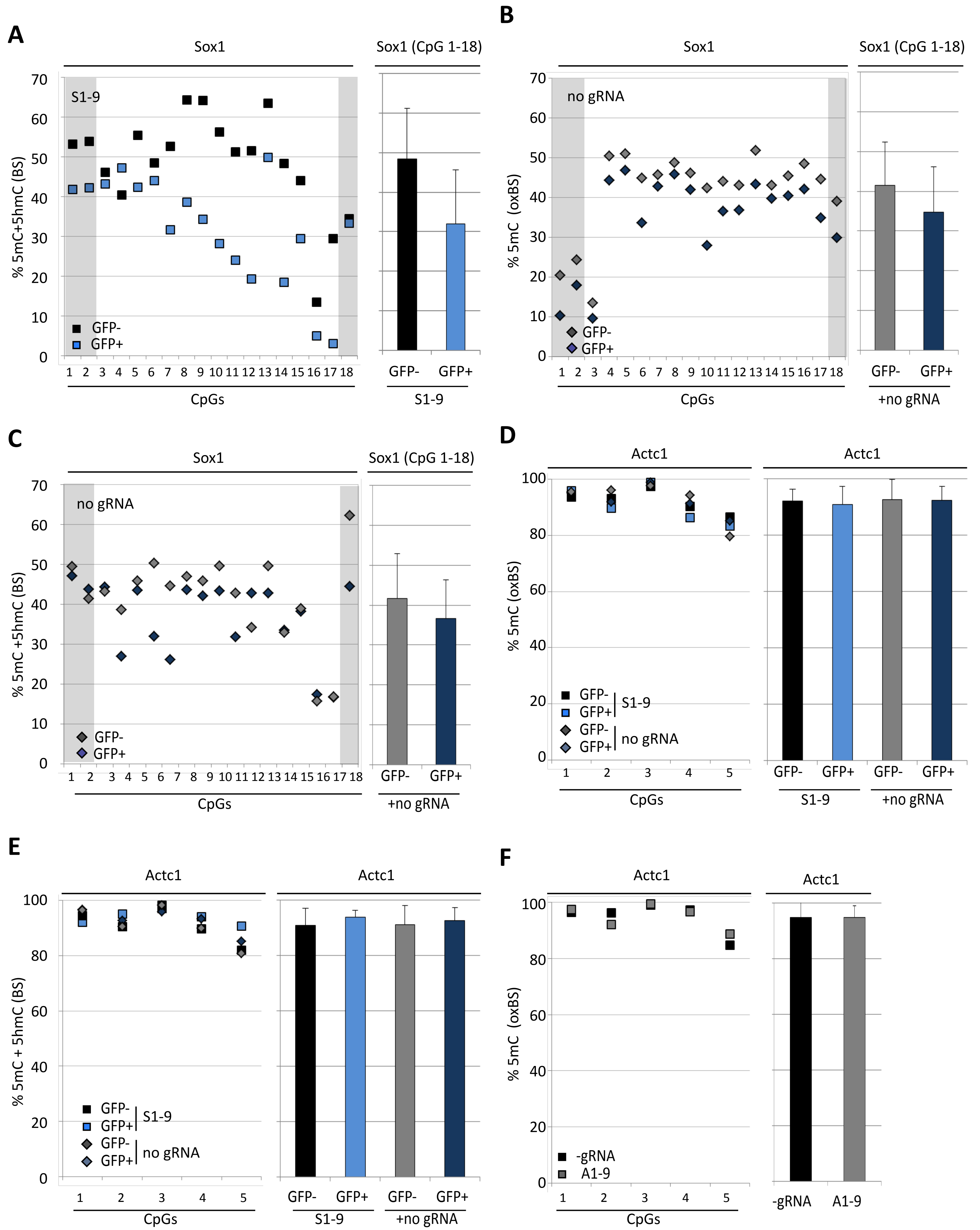

A
Sox1
Sox1 (CpG 1-18)
S1-9
% 5mC+5hmC (BS)
GFP-
GFP+
CpGs
S1-9
B
Sox1
Sox1 (CpG 1-18)
no gRNA
% 5mC (oxBS)
GFP-
GFP+
CpGs
+no gRNA
C
Sox1
Sox1 (CpG 1-18)
no gRNA
% 5mC +5hmC (BS)
GFP-
GFP+
CpGs
+no gRNA
D
Actc1
Actc1
% 5mC (oxBS)
GFP- GFP+ S1-9
GFP- GFP+ no gRNA
CpGs
S1-9
+no gRNA
E
Actc1
Actc1
% 5mC + 5hmC (BS)
GFP- GFP+ S1-9
GFP- GFP+ no gRNA
CpGs
S1-9
+no gRNA
F
Actc1
Actc1
% 5mC (oxBS)
-gRNA
A1-9
CpGs

Supplementary Figure 5

(A) Bisulfite sequencing corresponding to oxidative bisulfite sequencing in Figure 3G. Data shown as the mean (dot plot) and mean and standard error of the mean of all CpGs (bar graph) of n = 2 biological replicates of experiments performed on different days.

(B) Oxidative bisulfite and (C) bisulfite sequencing of sorted cell populations without gRNAs. No differences in DNA methylation and/or hydroxyl-methylation were apparent.

(D+E) No significant changes in DNA methylation and/or hydroxyl-methylation were seen at a control locus (*Actc1* promoter) when *Sox1* was targeted with dCas9-VP64 and/or when *Sox1*^{GFP}-positive and -negative fractions were analyzed, indicating no global changes in DNA modifications in these populations. Data shown as the mean (dot plot) and mean and standard error of the mean of all CpGs (bar graph) of n = 2 biological replicates of experiments performed on different days.

(F) No differences in DNA methylation at the *Actc1* promoter were detected after the transcriptional induction of *Actc1*. After transfection of gRNAs targeting *Actc1*, oxidative bisulfite sequencing was performed on transfected and untransfected cells. No difference in the level of DNA methylation could be observed, indicating that transcriptional engineering does not automatically result in local DNA de-methylation. Data shown as the mean (dot plot) and mean with standard error of the mean of all analyzed CpGs (bar graph) from n = 2 biological replicates. Analysis was performed on different days.

Supplementary Figure 6

Baumann et al.

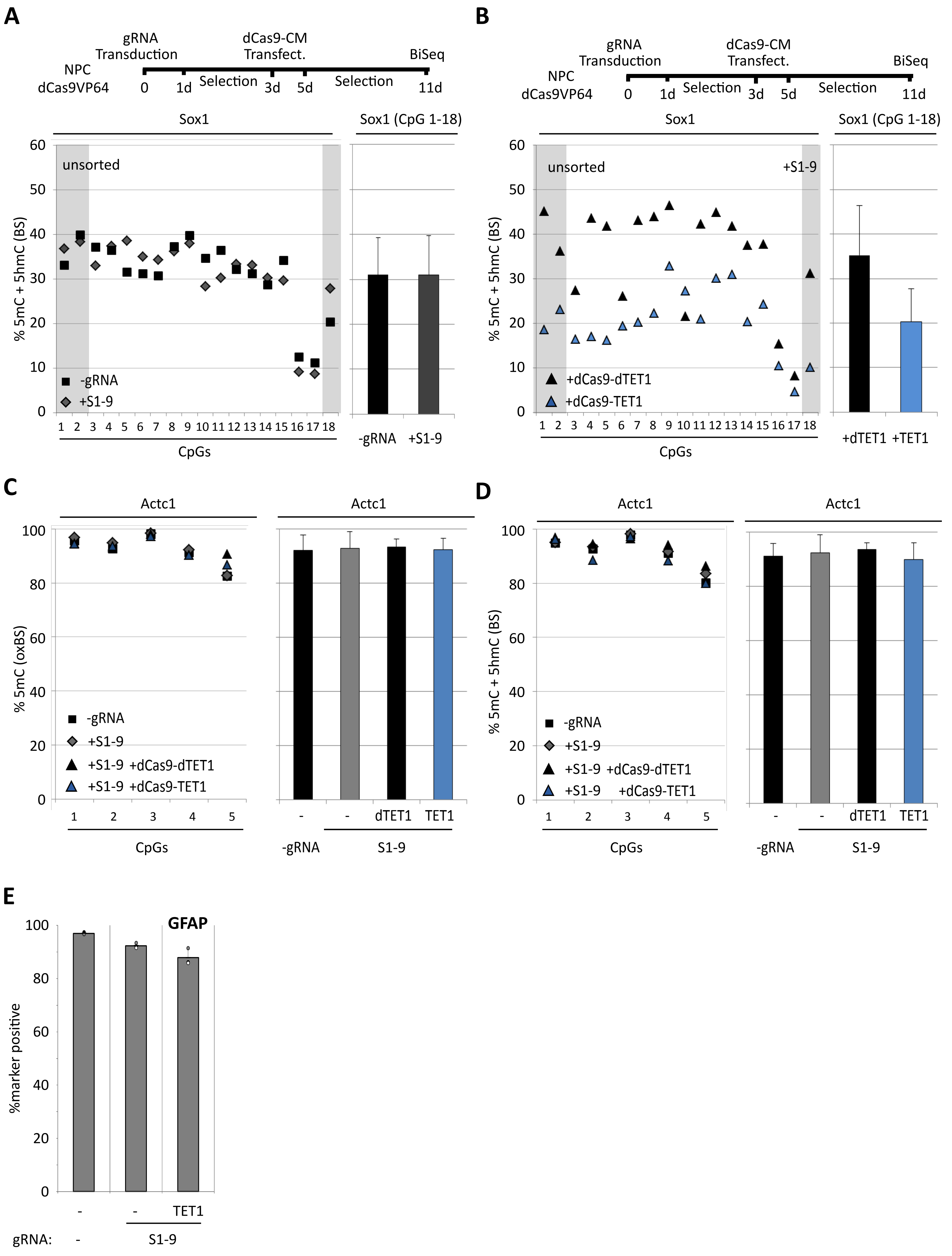

Supplementary Figure 6:

(A+B) Bisulfite sequencing corresponding to oxidative bisulfite sequencing in Figures 5 C and D. Data shown as the mean (dot plot) and mean and standard error of the mean of all CpGs (bar graph) of n = 4 biological replicates of experiments performed on different days.

(C+D) DNA methylation at the *Actc1* locus was analyzed in all samples shown in Figure 4C and D as an intrinsic control. No significant differences in DNA methylation and/or hydroxyl-methylation were detected.

(E) Differentiation assays revealed changes in the potency of SoxI-positive NPCs after DNA de-methylation. Cells were differentiated for 7 days; the number of cells positive for the glial marker Gfap has been quantified (n = 3 biological replicates, performed and analyzed independently on different days).

Supplementary Tables

Supplementary Table 1: Primer Sequences

**Primer name**	**Sequence**
Hygro_1fwd	AGTCAATAATCAATGTCAACCGGGTAGGGGAGGCG
Hygro_1rev	GGTGGGCGAAGAACTCTCGGCATCTACTCTATTCCTTTG
Hygro_2fwd	GAATAGAGTAGATGCCGAGAGTTCTTCGCCCACCCC
Hygro_2rev	AAGTGCCACCTGACGTCGACGGGTATACAGACATGATAAGATACATTGATGA
P300_fwd	CGATGACAAGGCTGCAGGAGGCGGAGGTAGCAAAGAAAATAAGTTTTCTGC TAAAAGG
P300_rev	GCTGATCAGCGGGTTTTCAGCATTCATTGCAGGTGTAGACAAA
Set7_fwd	CGATGACAAGGCTGCAGGAGGCGGAGGTAGCTTCTTCTTTGATGGCAGCACC
Set7_rev	GCTGATCAGCGGGTTTTCACTTTTGCTGGGTGGCC
Tet1_fwd	CGATGACAAGGCTGCAGGAGGCGGAGGTAGCGAACTGCCCACCTGCAGCTG
Tet1_int_rev	GGCAGTGACGAAGGCTTACT
Tet1_int_fwd	AGTAAGCCTTCGTCACTGC
Tet1_rev	GCTGATCAGCGGGTTTTCAGACCCAATGGTTATAGG
JMJD2a_fwd	CGATGACAAGGCTGCAGGAGGCGGAGGTAGCGCTTCTGAGTCTGAAACTCTG AATCC
JMJD2a_rev	GCTGATCAGCGGGTTTTCATGCTTCTGGCGTGGGCAG
Tet1_mut_fwd	GTGCTCATCCCTACAGGGCCATTCACAACAT
Tet1_mut_rev	ATGTTGTGAATGGCCCTGTAGGGATGAGCAC
dCas9-lenti-T2A-puro_fwd	ATTTCAGGTGTCGTGACGTACGGCCACCATGGATAAAAAGTATTCTATTGGTT TAG
dCas9-lenti-T2A-puro_rev	GCCCTCTCCACTGCCTGTACAGTTAATTAACATATCGAGATCGAAATCG
amp_gRNA_pLKO_fwd	CCATTCGATTAGTGAACGGATC

amp_gRNA_rev	CGACTCGGTGCCACTTTTTC
libgen_fwd	CTTGTGGAAAGGACGAAACA
libgen_rev	GCCTTATTTTAACTTGCTATTTCTAGC
Sox1_BiS_fwd chr8:12395267	TCGTCGGCAGCGTCAGATGTGTATAAGAGACAGTTTTGGGTTTTTAATTTAAT
Sox1_BiS_rev chr8:12395400	GTCTCGTGGGCTCGGAGATGTGTATAAGAGACAGAACCACACTACTTCCTAAAC
ActcI_BiS_fwd chr2:114052775	TCGTCGGCAGCGTCAGATGTGTATAAGAGACAGGTTGGATTTAGTTGGGTTGG
ActcI_BiS_rev chr2:114052908	GTCTCGTGGGCTCGGAGATGTGTATAAGAGACAGATAACAAATCAAAAACCCCC
Oct4_BiS_1_fwd Chr17:35505654	TCGTCGGCAGCGTCAGATGTGTATAAGAGACAGGGAGTGGTTTTAGAAATAATTGG
Oct4_BiS_1_rev Chr17:35505825	GTCTCGTGGGCTCGGAGATGTGTATAAGAGACAGCACCCCTACCTTAAATCAC
Oct4_BiS_2_fwd Chr17:35505829	TCGTCGGCAGCGTCAGATGTGTATAAGAGACAGGGTGAGAGGATTTTGAAGGT
Oct4_BiS_2_rev Chr17:35506148	GTCTCGTGGGCTCGGAGATGTGTATAAGAGACAGAAAAACAAAACTATAAAAATAAAAA
Nkx2-2_BiS_1_fwd Chr2: 147186667	TCGTCGGCAGCGTCAGATGTGTATAAGAGACAGTTTTTAGAGTAAGATGAGAGGTG
Nkx2-2_BiS_1_rev Chr2: 147186882	GTCTCGTGGGCTCGGAGATGTGTATAAGAGACAGATATTAAAAAAAATTCTTTACCCCC
Nkx2-2BiS_2_fwd Chr2: 147186852	TCGTCGGCAGCGTCAGATGTGTATAAGAGACAGATAGAAAGGAGGGGGTAAAGAATTT
Nkx2-2_BiS_2_rev Chr2: 147187043	GTCTCGTGGGCTCGGAGATGTGTATAAGAGACAGTAAATCTTATTTAAAAAACCACCAA
NeuroD4_BiS_fwd chr10:130280852	TCGTCGGCAGCGTCAGATGTGTATAAGAGACAGGGAGGGGTTATTTTGTGGGTA
NeuroD4_BiS_rev chr10:130281135	GTCTCGTGGGCTCGGAGATGTGTATAAGAGACAGCACTACCAAAATACCTTC

	ATATCAATAC
Ngn2_BiS_1_fwd chr3:127632608	TCGTCGGCAGCGTCAGATGTGTATAAGAGACAGTAATGAGTTGTTGAAAGGGAG
Ngn2_BiS_1_rev chr3:127632690	GTCTCGTGGGCTCGGAGATGTGTATAAGAGACAGCAACTAACCAATCAATATTCC
Ngn2_BiS_2_fwd chr3:127632872	TCGTCGGCAGCGTCAGATGTGTATAAGAGACAGTGATTAGATAAAGGGGGGA
Ngn2_BiS_2_rev chr3:127633074	GTCTCGTGGGCTCGGAGATGTGTATAAGAGACAGACCCCCTCCTCACCTACCCTT

Supplementary Table 2: Domain cloning

Domain	Aminoacids	Template	Primers used
Tet1 catalytic domain	1418-2136	Addgene plasmid 39454	Tet1_fwd<br>Tet1_int_rev<br>Tet1_int_fwd<br>Tet1_rev
JMJD2a	1-350	Addgene plasmid 38846	JMJD2a_fwd<br>JMJD2a_rev
Set7	52-366	Addgene plasmid 24082	Set7_fwd<br>Set7_rev
P300	1284-1673	Addgene plasmid 23252	P300_fwd<br>P300_rev

Supplementary Table 3: gRNA sequences

gRNA name	gRNA construct	Sequence
Sox1_1	S1-9	GTTAATCATTCGGAGCGCGC
Sox1_9	S1-9	GCGCGGGCGGCGGAGCAAGG
Sox1_4	S4-7	GAGGCAAAGGGGGCGAGCTC
Sox1_7	S4-7	GGGGGGGAACAAGGGCAGGA
ActcI_1	A1-9	GGCTCCAAGAATGGCCTCAG
ActcI_2	A1-9	GGGAGGGGCAGGCCAGCAAG

Sox1_1	SoxProm	GTTAATCATTCGGAGCGCGC
Sox1_2	SoxProm	GCGGGCGGGGAGAGGCAAAG
Sox1_3	SoxProm	GCGCGGGCGGGGAGAGGCAA
Sox1_4	SoxProm	GAGGCAAAGGGGGCGAGCTC
Sox1_5	SoxProm	GCCGCCGCGCGCGCGCTC
Sox1_7	SoxProm	GGGGGGGAACAAGGGCAGGA
Sox1_9	SoxProm	GCGCGGGCGGCGGAGCAAGG
Oct4_1	Oct4_STAgR	GGATGTTTTAGGCTCTCCAG
Oct4_2	Oct4_STAgR	CATCGCACCACAAAGCCTGT
Nkx2-2_1	Nkx2-2_STAgR	GCCCTCTAGAGCAAGATGAG
Nkx2-2_2	Nkx2-2_STAgR	TCCTTTGTATGTAAATACTG
NeuroD4_1	NeuroD4_STAgR	TGACTCTACTACCCTACATG
NeuroD4_2	NeuroD4_STAgR	GTGACTCTACTACCCTACAT
Ngn2_1	Ngn2_STAgR	CCACCAATCACAATAGACAG
Ngn2_2	Ngn2_STAgR	AGTGTTCCCGGGACTCCGGG

Supplementary Table 4: qPCR primer

primer	Sequence
Sox1_qPCR_fwd	AGACAGCGTGCCTTTGATTT
Sox1_qPCR_rev	TGGGATAAGACCTGGGTGAG
eGFP_qPCR_fwd	GAAGCAGCACGACTTCTTCAA
eGFP_qPCR_rev	AAGTCGATGCCCTTCAGCTC
Gapdh_qPCR_fwd	TTGCAGTGGCAAAGTGGAGA
Gapdh_qPCR_rev	CGTTGAATTTGCCGTGAGTG
Actcl_qPCR_fwd	ATGTGTGACGACGAGGAGAC
Actcl_qPCR_rev	CGGACAATTTCACGTTCAGCA
Gapdh_ChIP_fwd	ACCAGGGAGGGCTGCAGTCC
Gapdh_ChIP_rev	TCAGTTCGGAGCCCACACGC
Oct4_ChIP_fwd	CCCCAGGGAGGTTGAGAGTT

Oct4_ChIP_rev	AAGGGCTAGGACGAGAGGGA
Sox1_ChIP_fwd	GCTGAGCTGAGTGCAAAGTG
Sox1_ChIP_rev	CCCTGGGTCGTGTTTAAATG
Cas9_qPCR_fwd	TCGTAGGGACCGCACTCATT
Cas9_qPCR_rev	TCGCTTTTCGCGATCATCTT
Actc1_ChIP_fwd	GGCCATATAGGGAGCTAGGG
Actc1_ChIP_rev	AGAGCAATAAGCCCACTCCA

Supplementary Table 5: Antibodies

Antigen	Company	Catalog #	Application	Dilution
Sox1	abcam	ab87775	Western Blot	1:1000
			ICC	1:400
Actcl	proteintech	66125-1-1G	ICC	1:250
a-Tubulin	Sigma	T5168	Western Blot	1:2000
GFAP	Sigma	G3893	ICC	1:500
Tuj1	Sigma	T8660	ICC	1:1000
Cas9	Novus	NBP2-36440	Western Blot	1:500
Flag-M2	Sigma	F3165	ChIP	1:1000
H3K9me3	abcam	ab8898	ChIP	1:500
H3K27me3	Diagenode	C15410195	ChIP	1:1000
H3	abcam	ab1791	ChIP	1:1000
IgG	Diagenode	C15400001-15	ChIP	1:1000
NeuroD4	Novus	NBP2-13932	ICC	1:500
Nkx2-2	R&D Systems	MAB8162	ICC	1:500
Ngn2	R&D Systems	MAB3314	ICC	1:1000
Oct4	Abcam	Ab181557	ICC	1:300
E-Cadherin	Invitrogen	14-3249-82	ICC	1:300
Nestin	Millipore	MAB5326	ICC	1:500

Notch1	Invitrogen	MA5-11961	ICC	1:500
Occludin	Novus	NBP1-87402	ICC	1:250
Prominin	Biolegend	141201	ICC	1:300
Zo-1	Santa-Cruz	Sc-10804	ICC	1:500
Map2	Merck/Millipore	MAB378	ICC	1:300
vGlut1	Millipore	AB5905	ICC	1:300
Calbindin	Merck/Millipore	AB1778	ICC	1:1000

Supplementary Methods

qRT-PCR:

qRT-PCR primers can be found in Supplementary table 4. RNA from *in vitro* cultures was extracted using the RNeasy Mini Kit (Qiagen, 74104) according to manufacturer's protocol. RNA from tissue was extracted using the Extractme total RNA kit (DNAGDANSK, EM09.1) according to manufacturer's protocol. DNAse treatment and reverse transcription were performed with the Maxima first strand cDNA synthesis kit (ThermoFisher, K1671), and analyzed using PowerUp^{TM} Sybr Green Master Mix (ThermoFisher, A25742) on an Applied Biosystems™ QuantStudio™ 6 Flex Real-Time PCR System. Data are quantified as mRNA fold change normalized to *Gapdh*. Samples were analyzed in triplicates.

Lentivirus generation:

Lentiviral vectors were produced by transient co-transfection of HEK 293T cells with lentiviral packaging plasmid pCMVDR8.9, pseudotyping plasmid pVSVG and the lentiviral expression plasmid. 96 hours after transfection the supernatant was harvested, centrifuged at 4500 rcf for 10 minutes and filtered through a 0.45 µm filter (Millipore, SLHV033RS). Viral particles were pelleted at 27,000 rcf at 4°C for 2 hours, resuspended in TBS-5 (containing50 mM Tris-HCl (pH 7.8), 130 mM NaCl, 10 mM KCl, and 5 mM MgCl2) and stored at −80°C. Viral titers were measured for gRNA vectors (ca 10^8 infectious particles per ml on neural progenitor cells).

Flow cytometry and fluorescence activated cell sorting (FACS):

For flow analysis and cell sorting, cells were detached using Accutase (Sigma-Aldrich, A6964), washed twice with PBS and treated with eBioscience™ Fixable viability dye eFlour™ 660 (ThermoFisher, 65-0864-14) for 10 minutes at 37°C. Cells were analyzed and sorted in a FACSAriaIII™ (Becton Dickinson) flow cytometer. Cells were sorted at a flow rate of 5 (arbitrary units; corresponding to ~42µl/min) into cell culture medium supplied with 5% BSA and cultured under normal conditions immediately after sorting. The gating strategy is depicted in Supplementary Figure 1.

Western Blotting:

For western blot antibodies and PAGE gel concentrations see Supplementary Table 5. To extract proteins from cultured cells, cells were detached using Accutase and washed once in PBS. Proteins were extracted using RIPA buffer (SIGMA-ALDRICH, R0278) with 1x protease inhibitor cocktail. Protein concentrations were measured using a Bradford assay (Bio-Rad, 5000201). Around 40µg protein was loaded per well on a Polyacrylamide Protein Gel, separated by electrophoresis and blotted on a 0.2µm polyvinylidene membrane (ThermoFisher, LC2002) (activated with methanol). Membranes were incubated with the primary antibodies (concentrations see Supplementary Table 5), overnight at 4°C. Incubation with secondary antibodies and detection were performed according to routine laboratory practices. Uncropped blots are shown below.

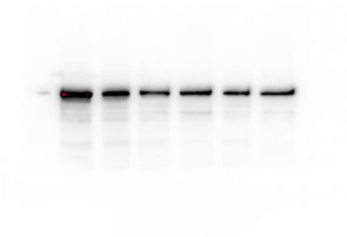

Uncropped Image of the dCas9 Western Blot

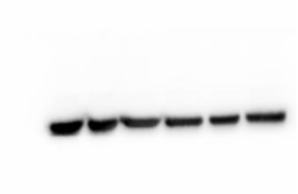

Uncropped Image of the loading control (alpha-Tubulin) for the dCas9 Western Blot

Uncropped Image of the Sox1 Western Blot

Uncropped Image of the loading control (alpha-Tubulin) for the Sox1 Western Blot

Differentiation assay:

To induce differentiation of NPCs, 40000 cells per well were plated on coverslips in 24-well plates. 3 wells were used per condition Cells were cultured in NeuroCult^{TM} Proliferation kit

supplemented with 1% (v/v) FCS and 1µg/ml Laminin, but without growth factors. Medium was exchanged every other day. After 7 days in differentiation condition, cells were fixed and immuocytochemistry was performed.

For assessment of neuronal subtype analysis, cells were differentiated for an additional 14 days. Starting at day 7, differentiation medium was supplemented with 10ng/ml NT3 (ThermoFisher Scientific, PHC7036), BDNF (Gibco, PHC7074), GDNF (Gibco, PHC7041), and cAMP (Sigma Aldrich, A6885), and exchanged the medium every other day. Cells were fixed after a total of 21 days of differentiation.

Immunofluorescence staining and microscopy analysis:

Primary and secondary antibodies are listed in Supplementary Table 5. For immunocytochemistry (ICC), cells were plated on PDL coated glass coverslips. Cells were fixed in 4% paraformaldehyde in PBS for 10 minutes at room temperature. Cells were first permeabilized in 0.5% Triton X-100 (Biomol, T8655.1) in PBS for 15 minutes at room temperature, followed by treatment in 3% BSA and 0.5% Triton X-100 in PBS for 30 minutes at room temperature. Cells were incubated in primary antibodies in 3% BSA (SIGMA-ALDRICH, A2153-1KG) and 0.5% Triton X-100 in PBS over night at 4°C. Cells were washed three times in PBS and incubated in appropriate secondary antibodies conjugated to fluorophores for 2 h in the dark at room temperature, followed by three washes in PBS and treatment with DAPI. Coverslips were mounted onto glass slides with Aqua-Poly/Mount (Polysciences, 18606-20). Stainings were analyzed using an epifluorescence microscope (Zeiss). Countings were performed on three randomly selected views per coverslip using a 10x objective. Pictures were analyzed using Fiji (National Institute of Health, USA,

RRID:SCR_002285) software. Values are calculated as mean of biological replicates. All samples of a biological replicate were treated equally and with linear image processing methods only.

RNA sequencing-library construction and sequencing:

Per condition, the RNA of 100000 cells of three independent biological samples was isolated using the PicoPure™ RNA Isolation Kit (ThermoFisher, KIT0204) according to manufacturer's protocol. The quality of RNA samples was determined with the Agilent Bioanalyzer 2100, accepting RNA integrity numbers (RIN) of >8. 4.5ng RNA per sample was used as input for cDNA synthesis. cDNA synthesis, amplification and purification of amplified cDNA was performed using the SMART-Seq™ v4 Ultra™ Low Input RNA Kit (TaKaRa, 634894) and Agencourt AMPure XP magnetic beads (Beckman Coulter, A63881) according to manufacturer's protocol. A total of 8 cycles was used for cDNA amplification. Quality of cDNA was validated using the Agilent Bioanalyzer 2100. Shearing and library preparation were performed according to the SMART-Seq™ v4 Ultra™ Low Input RNA Kit for Sequencing User Manual, using the Covaris S220 sonicator and the MicroPlex Library Preparation Kit v2 (Diagenode, C05010012). cDNA concentration and quality were assessed using the Agilent Bioanalyzer 2100. Samples were then pooled at equimolar amounts to a final concentration of 5nM. The cDNA library was sequenced by the Sequencing Core Facility of the Helmholtz-Zentrum München on a HiSeq4000 (Illumina). The sequences were aligned using the STAR v2.6 RNA-seq aligner (**RRID:SCR_015899**), expression calculated using RSEM v1.1.14 (**RRID:SCR_013027**) and analyzed using R Studio v 1.1.442 (**RRID:SCR_000432**). For comparative analysis, all transcripts were excluded that did not have at least 5 counts per

million in either the baseline or the SoxI positive NPC samples. The remaining genes were compared between the two conditions and significantly regulated genes selected according to an adjusted p-value <0.05 and an FDR value <0.01. Significantly regulated genes are listed in Supplementary Table7. From these genes, the 200 most upregulated and 200 most downregulated genes were selected. The expressional changes of those genes were calculated for each of the three samples of each experimental condition (Sox1 positive NPCs, NSCs, NRs), compared to the mean expression of the control group (baseline NPCs), and a heatmap was generated, based on these values. Raw and processed data has been uploaded to GEO (accession number GSE119480).

ChIP-qPCR:

ChIP-grade antibodies and qPCR primers are listed in Supplementary Table 5 and Supplementary Table 4respectively. To extract Chromatin for immunoprecipitation, $5x10^6$ cells were washed twice with PBS and fixed for 8 minutes at room temperature in 1% methanol free formaldehyde (ThermoFisher, 28906) in PBS. Formaldehyde was quenched by addition of 57µl 1.25M glycine and incubation for 5 minutes at room temperature. Cells were washed twice with cold PBS and incubated in hypotonic buffer (containing 10mM Tris-Hcl, 2mM MgCl2, 0.5% Triton X-100 and 1x Protease inhibitor cocktail) for 10 minutes on ice. After centrifugation at 500g and 4°C for 5 minutes, nuclei were resuspended in lysis buffer (containing 1% (v/v) SDS, 10mM EDTA, 50mM Tris-Hcl, and 1x Protease inhibitor cocktail) and incubated on ice for 30 minutes. Chromatin was sheared with the Bioruptor™ (Diagenode), using 5 cycles of 30 seconds on and 30 seconds off. Chromatin immunoprecipitaion and purification of genomic DNA was performed on the IP-Star®

Compact (Diagenode) employing the Auto iDeal ChIP-seq kit for Histones (Diagenode, C01010171) according to manufacturer's protocol. Samples were analyzed in triplicates using PowerUp™ Sybr Green Master Mix on an Applied Biosystems™ QuantStudio™ 6 Flex Real-Time PCR System, and quantified over the input sample.

Bisulfite and oxidative bisulfite Sequencing:

Primers for library preparation are listed in Supplementary Table6. Genomic DNA was extracted from 200000 cells per sample using the DNeasy Blood and Tissue kit (Quiagen, 69504) and DNA content was measured with a NanoDrop. For analysis of the total amount of methylation and hydroxymethylation, bisulfite conversion was performed directly after DNA preparation from genomic DNA. For analysis of DNA methylation in specific, oxidation with $KRuO_4$ was performed prior to conversion.

For oxidation, 1µg DNA was denatured in 0.05M NaOH (Sigma Aldrich, 1310-73-2) at 37°C for 30 minutes. During incubation, Micro-Bio-Spin P-6 SCC columns (Bio-Rad, 7326201) were washed four times by each time adding 500µl H_2O, centrifugation at 1000 x rcf for 1 minute, and discarding the flow-through. After denaturation, DNA was loaded on a prepared Micro-Bio-Spin column and centrifuged for 8 minutes at 1000 x rcf. DNA was snap cooled and then oxidized for 1 hour with 15mM $KRuO_4$ (Sigma Aldrich, 10378-50-4) in 0.05M NaOH. DNA was then purified by loading on a prepared Micro-Bio-Spin column and centrifugation for 8 minutes at 1000 x rcf.

The DNA was bisulfite converted using the EZ DNA Methylation-Gold Kit (Zymo, D5005) and the resulting modified DNA immediately used as template for the following PCR. The loci of interest were amplified by PCR, using HotStar Taq (Qiagen, 203203). The PCR mix contained 2.5µl of 10x buffer, 1µl of 10nM dNTPs, 0.25µl per primer, 100ng DNA, and 0.5µl Taq Polymerase. The PCR products were purified using Agencourt AMPure XP magnetic beads according to manufacturer's protocol. To attach the Illumina sequencing adapters, an adapter PCR was performed, using primer sequences from the Nextera DNA Library Preparation Kit (Illumina, FC-121-1012). The PCR mix contained 1µl DNA, 0.75µl of each indexing primer, 12.5µl of Phusion® High-Fidelity PCR Master Mix, and 10µl water. Samples were purified using Agencourt AMPure XP magnetic beads according to manufacturer's protocol. Samples were pooled and analysed at the Agilent Bioanalyzer 2100. The library pool was diluted to 5nM and sequenced by the sequencing core facility of the Helmholtz-Zentrum München on a HiSeq4000 (Illumina). The sequences were aligned and analyzed with the Bismark Bisulfite Mapper v0.15.0 (Babraham Institute, **RRID:SCR_005604**). Raw and processed data has been uploaded to SRA (accession numbers PRJNA490128, PRJNA522700, and PRJNA522707).
