# Supplementary material for: Targeted removal of epigenetic barriers during transcriptional reprogramming
Source: Nat Commun. 2019 May 9;10:2119. doi: 10.1038/s41467-019-10146-8 (PMC6509258; doi:10.1038/s41467-019-10146-8)
Supplement: Supplementary file 3 — Reporting Summary [file 41467_2019_10146_MOESM3_ESM.pdf]

nature research

Corresponding author(s): Dr. Stefan H. Stricker

Last updated by author(s): Apr 9, 2019

Reporting Summary

Nature Research wishes to improve the reproducibility of the work that we publish. This form provides structure for consistency and transparency in reporting. For further information on Nature Research policies, see Authors & Referees and the Editorial Policy Checklist.

Statistics

For all statistical analyses, confirm that the following items are present in the figure legend, table legend, main text, or Methods section.

n/a	Confirmed	
☐	☒	The exact sample size (*n*) for each experimental group/condition, given as a discrete number and unit of measurement
☐	☒	A statement on whether measurements were taken from distinct samples or whether the same sample was measured repeatedly
☐	☒	The statistical test(s) used AND whether they are one- or two-sided<br>*Only common tests should be described solely by name; describe more complex techniques in the Methods section.*
☒	☐	A description of all covariates tested
☒	☐	A description of any assumptions or corrections, such as tests of normality and adjustment for multiple comparisons
☐	☒	A full description of the statistical parameters including central tendency (e.g. means) or other basic estimates (e.g. regression coefficient) AND variation (e.g. standard deviation) or associated estimates of uncertainty (e.g. confidence intervals)
☐	☒	For null hypothesis testing, the test statistic (e.g. *F*, *t*, *r*) with confidence intervals, effect sizes, degrees of freedom and *P* value noted<br>*Give P values as exact values whenever suitable.*
☒	☐	For Bayesian analysis, information on the choice of priors and Markov chain Monte Carlo settings
☒	☐	For hierarchical and complex designs, identification of the appropriate level for tests and full reporting of outcomes
☒	☐	Estimates of effect sizes (e.g. Cohen's *d*, Pearson's *r*), indicating how they were calculated

Our web collection on statistics for biologists contains articles on many of the points above.

Software and code

Policy information about availability of computer code

Data collection	n.A.
Data analysis	Data was analysed using RStudio

For manuscripts utilizing custom algorithms or software that are central to the research but not yet described in published literature, software must be made available to editors/reviewers. We strongly encourage code deposition in a community repository (e.g. GitHub). See the Nature Research guidelines for submitting code & software for further information.

Data

Policy information about availability of data

All manuscripts must include a data availability statement. This statement should provide the following information, where applicable:

- Accession codes, unique identifiers, or web links for publicly available datasets
- A list of figures that have associated raw data
- A description of any restrictions on data availability

Raw Sequencing data is available under accession numbers GSE119480 (RNAseq) and PRJNA490128, PRJNA522700, and PRJNA522707 (Bisulfite and oxidative Bisulfite Sequencing). Raw Data underlying all Figures are provided as a Source Data File. All relevant data can also be inquired from the authors.

Field-specific reporting

Please select the one below that is the best fit for your research. If you are not sure, read the appropriate sections before making your selection.

☒ Life sciences ☐ Behavioural & social sciences ☐ Ecological, evolutionary & environmental sciences

Life sciences study design

All studies must disclose on these points even when the disclosure is negative.

Sample size	No sample size calculation was performed beforehand. Sample sizes were chosen according to established practice in the field.
Data exclusions	No data point has been excluded.
Replication	For reproducibility biological replicates were performed on different days and different clonal cell lines.
Randomization	n/a
Blinding	n/a

Reporting for specific materials, systems and methods

We require information from authors about some types of materials, experimental systems and methods used in many studies. Here, indicate whether each material, system or method listed is relevant to your study. If you are not sure if a list item applies to your research, read the appropriate section before selecting a response.

Materials & experimental systems

n/a	Involved in the study	
☐	☒	Antibodies
☐	☒	Eukaryotic cell lines
☒	☐	Palaeontology
☒	☐	Animals and other organisms
☒	☐	Human research participants
☒	☐	Clinical data

Methods

n/a	Involved in the study	
☒	☐	ChIP-seq
☐	☒	Flow cytometry
☒	☐	MRI-based neuroimaging

Antibodies

Antibodies used	see Supplementary Table 5
Validation	only commercial and validated antibodies have been used.

Eukaryotic cell lines

Policy information about cell lines

Cell line source(s)	NPCs have been derived from Sox1-GFP ESCs (Prof.Austin Smith).
Authentication	Genotype have been authenticated (PCR), dCas9 clones have been authenticated (Immunoblot, PCR)
Mycoplasma contamination	Cells were not tested for Mycoplasma
Commonly misidentified lines (See ICLAC register)	no commonly misidentified lines have been used

Flow Cytometry

Plots

Confirm that:

☒ The axis labels state the marker and fluorochrome used (e.g. CD4-FITC).

☒ The axis scales are clearly visible. Include numbers along axes only for bottom left plot of group (a 'group' is an analysis of identical markers).

☒ All plots are contour plots with outliers or pseudocolor plots.

☒ A numerical value for number of cells or percentage (with statistics) is provided.

Methodology

Sample preparation	Detachment (Accutase) and stained for viability dye as described in the Methods
Instrument	FACS Aria III (Becton Dickinson)
Software	FloJo X 10.0.7r2
Cell population abundance	Sorting has been performed under "purity" setting.
Gating strategy	FSC-A vs FSC-W (singlets), FSC-A vs SSC-A (debris exclusion), FSC-A vs 660-20 (live cells)

☒ Tick this box to confirm that a figure exemplifying the gating strategy is provided in the Supplementary Information.
